# Development and validation of a nomogram based on common biochemical indicators for survival prediction of children with high-risk neuroblastoma: A valuable tool for resource-limited hospitals

**DOI:** 10.1186/s12887-023-04228-2

**Published:** 2023-08-26

**Authors:** Ruohao Wu, Xiaohui Li, Zhishan Chen, Qiong Shao, Xiao Zhang, Wenting Tang, Bo Hu

**Affiliations:** 1 Department of Pediatrics, Sun Yat-Sen Memorial Hospital, Sun Yat-Sen University, Guangzhou, 510120 Guangdong China; 2 Department of Laboratory Medicine, Sun Yat-Sen University Cancer Center, Sun Yat-Sen University, Guangzhou, 510060 Guangdong China; 3 Department of Pathology, Panyu District Central Hospital, Guangzhou, 511400 Guangdong China; 4 Department of Research and Molecular Diagnostics, Sun Yat-Sen University Cancer Center, Sun Yat-Sen University, Guangzhou, 510060 Guangdong China; 5 Department of Laboratory Medicine, Third Affiliated Hospital, Sun Yat-Sen University, Guangzhou, 510630 Guangdong China

**Keywords:** Ultra high-risk neuroblastoma, Nomogram, Pretreatment, Lactate dehydrogenase, Albumin, Overall survival, Developing country/region

## Abstract

**Background:**

Despite multiple attempts have been made to develop risk stratification within high-risk neuroblastoma (NB) patients (age of diagnosis ≥ 18 month-old with metastatic NB), the definition of “ultra high-risk NB” is still lack of consensus, and indicators for identifying this subgroup are still unclear. This study aimed to develop a nomogram based on easy-to-obtain blood-derived biofactors for identifying ultra high-risk NB patients with highest risk of death within 3 or 5 years.

**Methods:**

One hundred sixty-seven NB patients who treated at Sun Yat-sen University Cancer Center between 2015 and 2023 were recruited and clustered randomly into training and validation cohorts (116 and 51 cases, respectively). Univariate and multivariate Cox analysis were performed in training set to screen independent prognostic indicators for constructing nomogram model of predicting 1-, 3- and 5-year overall survival (OS). The discrimination power of the nomogram in training and validation sets were assessed by concordance index (C-index) and calibration plot. Based on the risk score obtained from nomogram model, the prognostic accuracy of 1-, 3- and 5-year OS rates in training and validation cohorts were further evaluated using the area under receiver operating characteristic (ROC) curves (AUC).

**Results:**

Through univariate and multivariate Cox analysis, independent prognostic indicators, including serum lactate dehydrogenase (LDH) and albumin (ALB), were identified in training set, and used to establish a nomogram model. The model showed good discrimination power with C-index in training cohort being 0.706 (95%CI: 0.633—0.788). According to the cut-point calculated based on the established nomogram, patients with a nomogram score > 34 points could be stratified to ultra high-risk NB subgroup, and this subgroup had poorer OS than those in non-ultra one (*p* < 0.001). AUC values of ROC curves for 3- and 5-year OS rates in the training set were 0.758 and 0.756, respectively. Moreover, based on the cut-point score (34 points) developed in training set, The model also showed good discrimination power with C-index of 0.773 (95%CI: 0.664—0.897) and powerful prognostic accuracy of AUC for 3- and 5-year OS rates being 0.825 and 0.826, respectively, in validation cohort.

**Conclusions:**

We developed a simple-to-use nomogram based on common laboratory indicators to identify the subgroup of ultra high-risk NB before treatment, providing these children even from developing countries or regions access to intensified multimodal treatments earlier and thus improving their long-term outcome.

**Supplementary Information:**

The online version contains supplementary material available at 10.1186/s12887-023-04228-2.

## Introduction

Neuroblastoma (NB) originates from the sympathetic nerve cells and can occur in any parts of the sympathetic nervous system, including adrenal glands, pelvis, abdomen, chest and neck. NB also is one of the most common types of extracranial solid tumors in children, which constitutes about 11% of pediatric tumors [1, 2]. The prognosis of NB is quite variant; patients with low-risk NB (age at diagnosis < 18 months or non-metastatic NB) generally have a favorable prognosis and merely require intensified anti-cancer treatments, while children with high-risk NB (age at diagnosis ≥ 18 months with metastatic NB) mostly show an unfavorable prognosis, regardless of improvements in multimodal anti-NB therapy, including surgery, chemotherapy, radiotherapy, hematopoietic stem cell transplantation and immunotherapy [3], with 3-year and 5-year overall survival (OS) rates being 60% [4] and 50% [5], respectively. In China, the 3-year and 5-year OS rates are often lower than 60% and 50% owing to some therapeutic limitations and racial differences. For instances, the CAR-T therapy and anti-GD2 immunotherapy are often hard to obtain and costly in China; moreover, the OS rates of Asian NB patients are reported to be lower than that of black and white ones [1]. With the refined stratification definition for low-risk NB patients putting into effect, "middle low-risk NB" subgroup could be well-identified from low-risk NB group [6], the prognosis of children with high-risk NB still remained heterogeneous [7], and there is lack of agreement on the term of “ultra high-risk NB” (a subgroup that has the highest risk of death within 3 or 5 years). Thus, it is necessary to develop a refined stratification system for early screening the subgroup of “ultra high-risk NB” at the time of diagnosis.

Currently, there are two main stratification systems for NB, *i.e.*, International Neuroblastoma Staging System (INSS) [8] and International Neuroblastoma Risk Group Staging System (INRGSS) [9]. However, INSS and INRGSS are both mainly used for guiding the intensity of the anti-NB therapy, they are unable to help in predicting the OS of high-risk NB individually at the time of diagnosis. On the basis of INSS and INRGSS, Children’s Oncology Group (COG) risk stratification system, including age of diagnosis, *MYCN* status, DNA ploidy, tumor histologic classification and INSS/INRGSS stages, was following established [10]. However, this risk stratification system is used for all risk types of NB, there is no specific stratification system solely used for high-risk NB group to identify “ultra high-risk NB” children with the poorest outcome. On the other hand, although many researchers using novel omic-related technologies to develop stratification systems for high-risk NB group, none of them have been validated clinically and applied for clinical use [11].

Nomogram is a powerful predictive model and widely used in forecasting outcomes of many human cancers due to its objectivity, accuracy and visualization [12]. To date, some nomograms using NB prognosis-related biomarkers, such as age at diagnosis*, MYCN* status, DNA ploidy and tumor histology, have been already reported in NB [1–3]. However, there is only one prognostic nomogram applied specifically for high-risk NB, this study based on the International Neuroblastoma Risk Group (INRG) public database reported a nomogram for the prognosis prediction of 3-year OS in high-risk NB patients [3]. However, the study was only used for predicting 3-year OS, and only incorporated patients enrolled on trials at Europe, United States and Japan. To the best of our knowledge, there is no prognostic nomogram reported concretely based on Chinese high-risk NB population or applied to predict 1-, 3- and 5-year OS rates for high-risk NB children from developing countries or regions. Thus, we were preparing this article, a single-center study based on Chinese population generated a user-friendly prognostic nomogram for high-risk NB. We took advantage of these easily obtainable and common biochemical blood-derived indicators, developing a nomogram model especially for developing countries or regions to evaluate the precise risk in high-risk NB children.

## Methods

### Patients

This retrospective research consisted of 167 Chinese pediatric patients with high-risk NB, who treated at Sun Yat-sen University Cancer Center during the period of March 2015 and March 2023. We collected clinical and laboratory baseline data from patients who met the following eligibility criteria: (a) individuals ≥ 18 months old (1.5 years old) at newly diagnosis with metastatic NB, *i.e.* INRGSS stage M or INSS stage 4, and excluded the presence of any other malignant disorders and any underlying diseases such as severe liver/kidney diseases or hematological disease; (b) individuals had not previously received any anti-cancer therapies or any anti-cancer related treatments, and had clear and complete baseline clinical and laboratory data. Enrolled children were randomly clustered into training cohort and validation cohort (116 and 51 pediatric patients, respectively) by using R packages "car" [13] and "survival" [14]. Our research was approved by the Hospital Ethical Committee of the Sun Yat-sen University Cancer Center (Approval Number: B2023-311–01), and the Hospital Ethical Committee of the Sun Yat-sen University Cancer Center approved a waiver of individual informed consent for this retrospective research.

Clinical baseline data, including age at diagnosis, gender, metastasis status (INRGSS/INSS stage) and overall survival (OS) data (survival status/fustat and survival time/futime), were collected. OS was defined as the time from date of diagnosis of high-risk NB to any form of death and the survival data were censored for individuals who were alive at the last follow-up. Laboratory baseline data, including the pretreatment biochemical biomarkers, including serum lactate dehydrogenase (LDH), serum albumin (ALB), albumin-to-globulin ratio (AGR), serum creatinine (CRE), peripheral white blood cell count (WBC), platelet count (PLT), lymphocyte count (Lym), neutrophil count (Neu) and neutrophil–lymphocyte ratio (NLR), were chose as the candidate prognostic indicators.

### Construction and evaluation of the nomogram

Independent prognostic indicators were screened via univariate and multivariate Cox regression analysis and then were applied to construct the nomogram model using the data from the training set for predicting 1-, 3- and 5-year OS rates. The discriminatory power of the nomogram model was assessed using calibration plot with concordance index (C-index) in the training and validation sets. Based on the cut-point score calculated according to the established nomogram, cases in training and validation cohorts were divided into non-ultra and ultra high-risk subgroups. Risk plots, including risk-expression heatmap and risk-survival status plot, and Kaplan–Meier curves were then used to further investigate the influences of nomogram stratification on OS between non-ultra and ultra high-risk NB subgroups. Area under the curve (AUC) values of receiver operating characteristic (ROC) curves visualized in R environment [15] was used to evaluate the prognostic accuracy of 1-, 3- and 5-year OS rates in training and validation cohorts. Moreover, The benefit of clinical application of the nomogram model under the reported 3- (< 60%) [4] and 5-year (< 50%) [5] OS rates of high risk NB in training and validation cohorts were also evaluated by using decision curve analysis (DCA).

### Statistical analysis

All statistical analysis and figures plotting were conducted with R environment [version 4.1.0] (http://www.R-project.org/). Survival curves were plotted through Kaplan–Meier survival method and compared via the log-rank test. The main R packages applied in this study included “Survival”, “foreign”, “rms”, “ggDCA”, “caret” and “survivalROC” [16]. All numerical data were presented as means ± standard error of mean (SEM), if not specifically noted. Results with *p* value < 0.05 were regarded as statistically significant throughout the study.

## Results

### Patient characteristics

A total of 167 high-risk NB pediatric patients were included in current study. about 69.5% of them (*n* = 116) were randomly selected as the training cohort for the development of the nomogram model, while the rest 30.5% children (*n* = 51) served as the validation cohort for the validation of the nomogram model. The baseline clinical characteristics and demographics of patients in training and validation sets were shown in Table 1, and the detailed information about those cases in training and validation cohorts could be found in Supplementary file 1 and Supplementary file 2, respectively. During the period of follow-up, 50 children died and 66 survived in the training cohort. Meanwhile, 14 pediatric patients died, and 37 pediatric patients survived in the validation cohort.

**Table 1:** Baseline demographics or characteristics of the children with newly diagnosed high-risk NB in our cancer center

Demographics or characteristic		Training cohort (*n* = 116) [means ± SEM/n(%)]	Validation cohort (*n* = 51) [means ± SEM/n(%)]	*P* value
Age at diagnosis (year)		4.48 ± 0.32	3.24 ± 0.32	0.076
Gender	Female	38 (32.76%)	20 (39.22%)	0.528
	Male	78 (67.24%)	31 (60.78%)	
Serum LDH (IU/L)		1065.21 ± 109.28	1203.29 ± 172.30	0.961
Serum ALB (g/L)		38.88 ± 0.45	40.99 ± 0.77	0.579
WBC count (10^9/L)		7.63 ± 0.28	8.30 ± 0.37	0.125
Neu count (10^9/L)		3.70 ± 0.21	3.51 ± 0.25	0.652
Lym count (10^9/L)		3.03 ± 0.17	3.97 ± 0.31	0.080
NLR		1.67 ± 0.14	1.28 ± 0.17	0.484
PLT count (10^9/L)		333.53 ± 13.94	352.57 ± 25.21	0.093
Serum CRE (µmol/L)		27.79 ± 0.96	22.92 ± 1.07	0.296
AGR		1.39 ± 0.04	1.60 ± 0.07	0.080
Survival status	Alive	66 (56.90%)	37 (72.55%)	0.081
	Dead	50 (43.10%)	14 (27.45%)	

*NB* neuroblastoma, *AGR* albumin-to-globulin ratio, *LDH* lactate dehydrogenase, *ALB* albumin, *PLT* platelet, *WBC* white blood cell, *CRE* creatinine, *Lym* lymphocyte, *Neu* neutrophil, *NLR* neutrophil-to-lymphocyte ratio

### Independent prognostic indicators selection

Univariate Cox analysis for all included biochemical indicators was performed in training cohort firstly to determine the potential survival-related biomarker. As shown in Table 2, the results of univariate Cox analysis revealed three candidate blood-derived markers (ALB, LDH, and PLT counts) had the potential prognostic value. Among them, high level of LDH was associated with poor OS of pediatric high-risk NB patients [HR (95%CI): 1.705 (1.353–2.149), *p* < 0.001]; while low values of ALB [HR (95%CI): 0.205 (0.055–0.767)] and PLT count [HR (95%CI): 0.595 (0.426–0.831)] were associated with high risk of death in high-risk NB children (all *p* < 0.05). Then, multivariate Cox analysis was performed by using these three biofactors. Finally, two survival-related markers (serum LDH and ALB) were identified to have independent prognostic value. Specifically, the risk of death in high-risk NB children with high level of LDH was higher than children with low level of LDH [HR (95%CI): 1.724 (1.354–2.197), *p* < 0.001]. Plus, the risk of death in pediatric high-risk NB patients with low level of serum ALB was also higher than pediatric high-risk NB patients with high level of serum ALB [HR (95%CI): 0.231 (0.060–0.885), *p* < 0.05] (Table 2). Thereafter, two variables (LDH and ALB) were confirmed as independent prognostic indicators in high-risk NB children for constructing the individualized prognostic nomogram model.

**Table 2:** Univariate and multivariate Cox analysis of OS in training cohort

Candidate indicators	HR (95%CI)	*P* value	Candidate indicators	HR (95%CI)	*P* value
ALB	0.205 (0.055–0.767)	0.019*	ALB	0.231 (0.060–0.885)	0.032*
AGR	0.404 (0.127–1.282)	0.124			
serum CRE	1.292 (0.710–2.352)	0.402			
LDH	1.705 (1.353–2.149)	6.19E-06***	LDH	1.724 (1.354–2.197)	1.02E-05***
Lym	0.693 (0.415–1.160)	0.163			
Neu	1.040 (0.669–1.616)	0.863			
NLR	1.185 (0.764–1.837)	0.449			
PLT	0.595 (0.426–0.831)	0.002**			
WBC	0.708 (0.408–1.226)	0.218			

*AGR* albumin-to-globulin ratio, *LDH* lactate dehydrogenase, *ALB* albumin, *PLT* platelet, *WBC* white blood cell, *CRE* creatinine; *Lym* lymphocyte, *Neu* neutrophil, *NLR* neutrophil-to-lymphocyte ratio, *OS* overall survival, *HR* hazard ratio

^*^ *p* < 0.05, ** *p* < 0.01, *** *p* < 0.001

### Construction of the prognostic nomogram

**Fig. 1:**
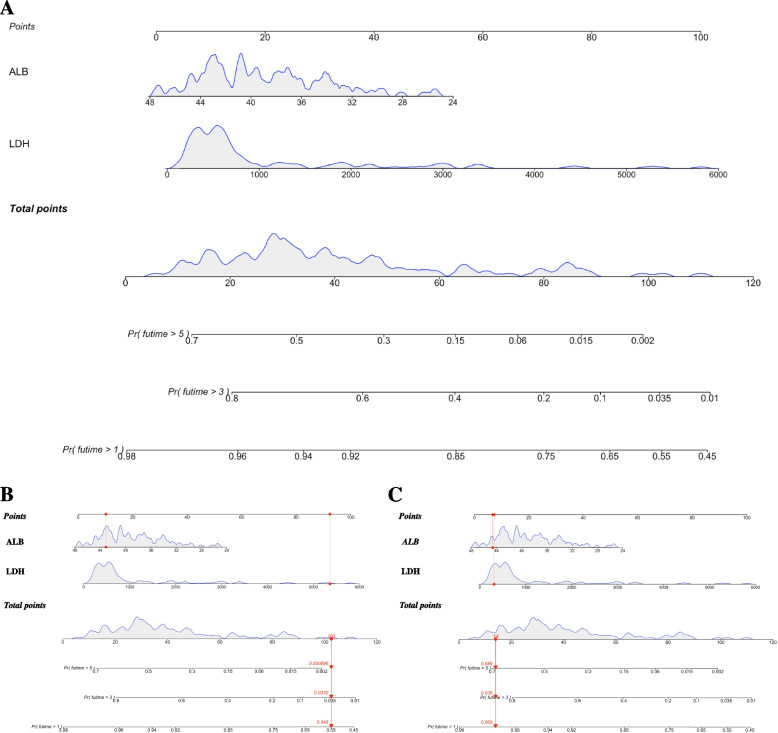
Nomogram for patients in training cohort at newly diagnosis with high-risk NB, estimating the probability of OS for 1-, 3- and 5-year. **A** The nomogram plot has two sections: the top section (starts with "Point" and goes down to the last factor, "LDH") is used to determine the number of scores for each included biofactor; while, the bottom section [starts with "Total Points" and goes down to "*Pr(futime* > *1)*"] is used to determine the probability of 1-, 3- and -year OS rates. The red arrows in (**B**) and (**C**) show two examples with different survival outcomes, respectively. Red arrow in (**B**): a pediatric high-risk NB patient with a total point of 103, the matched predicted probability of 1-, 3- and 5-year OS rates from the time of diagnosis are about 54.90%, 3.32% and 0.09%, respectively. Red arrow in (**C**), a high-risk NB child with a total point of 14, the matched predicted probability of 1-, 3-and 5-year OS rates from the time of diagnosis are about 96.90%, 83.50% and 68.90%, respectively. NB, neuroblastoma; OS, overall survival; LDH, lactate dehydrogenase; ALB, albumin

According to the results of final multivariate Cox regression analysis, LDH and ALB were incorporated into the prognostic nomogram model of high-risk NB. As shown in Fig. 1, the nomogram plot provided an estimate of the 1-, 3-, and 5-year OS probability [*Pr(futime* > *1)*, *Pr(futime* > *3)*, and *Pr(futime* > *5)*] using scored contributions of the levels of LDH and ALB, and could be used for estimating the probability of long-term survival for an individual child at the time of diagnosis. From the Fig. 1A, LDH had the greatest influence on OS rates, followed by serum ALB. A larger total points score meant a lower probability of 1-, 3- and 5-year OS rates. For instances, as demonstrated in Fig. 1B, a child with newly diagnosed high-risk NB (patient 67 in training cohort), regardless of gender, his/her serum level of LDH was 5356.8 IU/L (about 93 points) and serum ALB level was 43.1 g/L (about 10 points). Finally, the child received a total of 103 points indicating an estimated 1-, 3- and 5-year OS of approximate 54.90%, 3.32% and 0.09%, respectively. Actually, this child died after the diagnosis of 23 months (1.92 years). While another individual (patient 105 in training cohort) with LDH of 309.4 IU/L (about 8 points) and serum ALB of 44.6 g/L (about 6 points) had a total of 14 points indicating an estimated 1-,3- and 5-year OS of approximate 96.90%, 83.50% and 68.90%, respectively. In fact, this patient was still alive after the diagnosis of 89 months (7.42 years) (Fig. 1C).

### Evaluation of the nomogram discriminatory performance and prognostic accuracy in training cohort

**Fig. 2:**
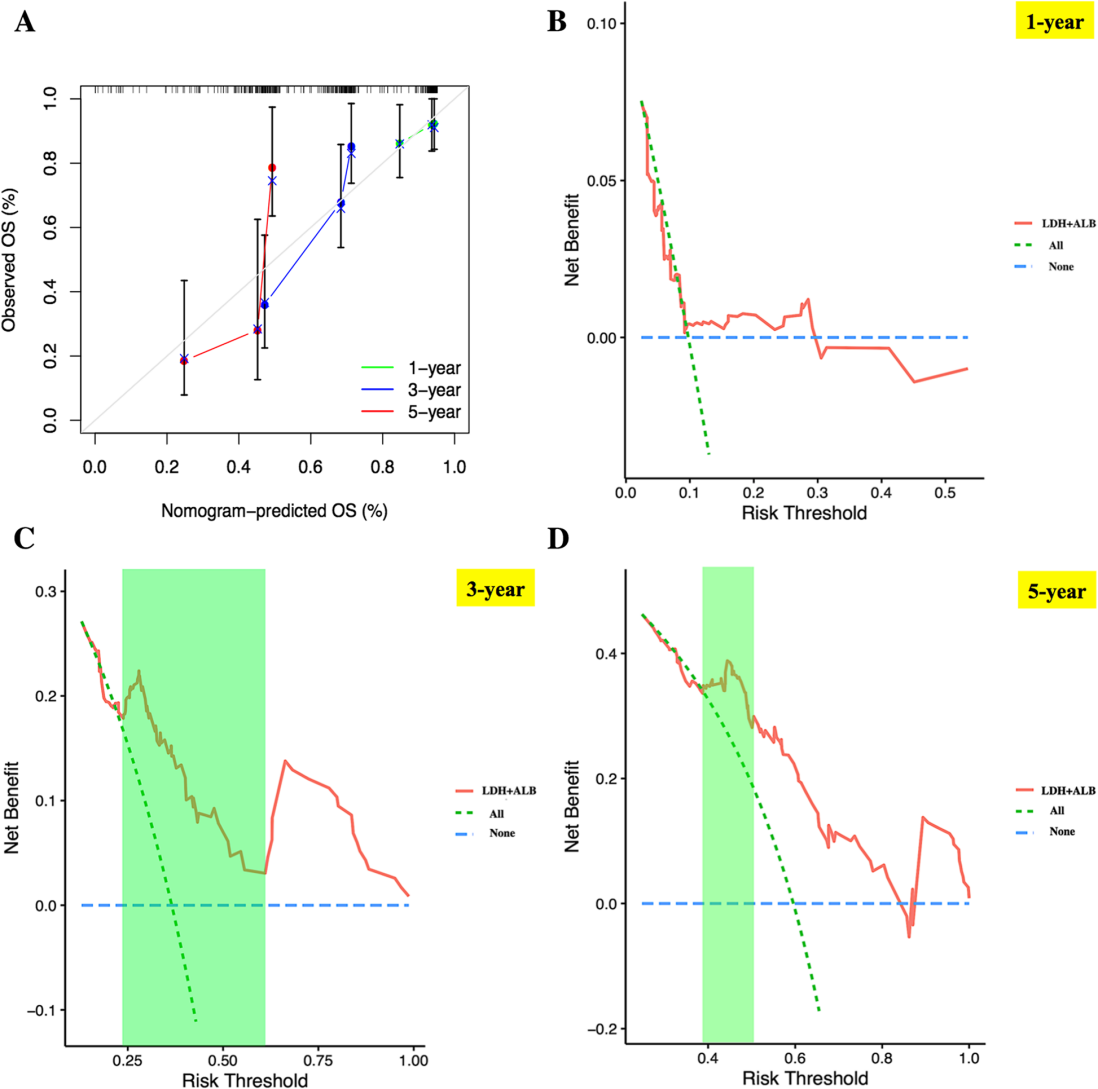
Nomogram discriminatory performance of the nomogram model in training cohort. **A** 1-, 3- and 5-year calibration plot assessing the estimation accuracy of the nomogram. **B**, **C** and **D** DCA curves examining the clinical utility of the nomogram model for 1-, 3- and 5-year probabilities of OS, respectively. It should be noted that for predicting the clinical utility of the model for 3- and 5-year OS rates, the clinical benefits were mainly determined by the sizes of area of right upper quadrant in green region. NB, neuroblastoma; OS, overall survival; DCA, decision curve analysis

**Fig. 3:**
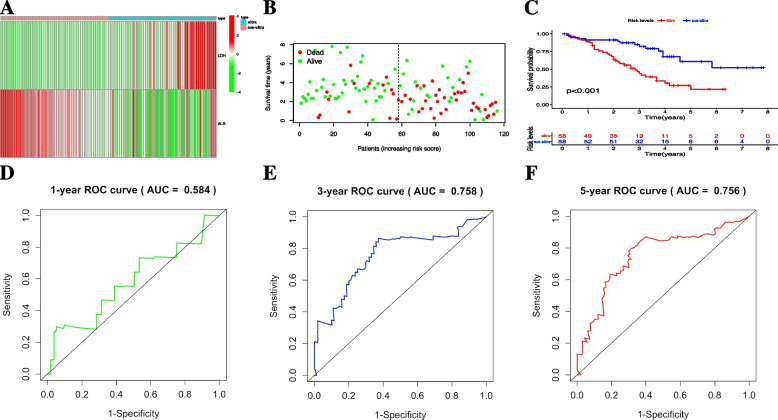
Prognostic accuracy of the nomogram model in training cohort. Prognostic evaluation of the nomogram model in training set based on the nomogram risk score, including (**A**) serum LDH/ALB level heatmap along with (**B**) survival time distribution plot and (**C**) the Kaplan–Meier curve between non-ultra high-risk and ultra high-risk groups. **D**, **E** and **F** ROC curves of the nomogram model for 1-,3-, and 5-year probabilities of OS. NB, neuroblastoma; OS, overall survival; LDH, lactate dehydrogenase; ALB, albumin; ROC, receiver operating characteristic

In training cohort, the discrimination power of nomogram model was firstly evaluated by using calibration plot with the value of C-index. The C-index of nomogram in training set was 0.706 (95%CI: 0.633—0.788). Meanwhile, calibration plot based on the nomogram model showed good consistency between actual 1-, 3- and 5-year OS and nomogram-predicted 1-, 3-, 5-year OS (Fig. 2A). Moreover, based on the risk score obtained from nomogram model for each individual in training set (Supplementary file 3), we applied decision curve analysis (DCA) to assess the clinical usefulness of the nomogram for predicting 1-, 3- and 5-year OS of high-risk NB in training cohort (Fig. 2B-D), and the result demonstrated that under the risk threshold of < 0.60 and < 0.50 (two reported overall survival rates of 3- and 5-year OS for high-risk NB children, respectively), children with high-risk NB could get more net benefit from this nomogram model than the hypothetical treat-none or treat-all scenarios, indicating using this nomogram to predict 3- and 5-year OS rates for high-risk NB survival may have more clinical benefit (area of right upper quadrant in green region in Fig. 2C, D). However, for predicting 1-year OS rate for these children, DCA curve didn't show much net benefit from nomogram compared with the hypothetical treat-none or treat-all scenarios, suggesting this nomogram may be not suitable for predicting 1-year OS rate (Fig. 2B). According to the median nomogram risk score (34 points) in training cohort, 116 pediatric patients were clustered into ultra high-risk (*n* = 58, nomogram score > 34 points) and non-ultra high-risk (*n* = 58, nomogram score ≤ 34 points) groups (Supplementary file 3). The heatmap of serum levels of LDH and ALB, and the distribution plot of survival time based on the median nomogram risk score demonstrated a good predictive value in training cohort (Fig. 3A and B); meanwhile, the Kaplan–Meier survival analysis was performed, and the result also demonstrated the ultra high-risk pediatric patients had significant worse OS than non-ultra high-risk pediatric patients in training set (*p* < 0.001, Fig. 3C). Finally, AUC values of ROC curves for 1-, 3- and 5-year OS rates in the training set were 0.584, 0.758 and 0.756, respectively, indicating that the nomogram model had a promising prognostic accuracy in the aspect of 3- and 5-year OS rates for children with high-risk NB (Fig. 3D-F).

### Validation of the nomogram discriminatory performance and prognostic accuracy in validation cohort

**Fig. 4:**
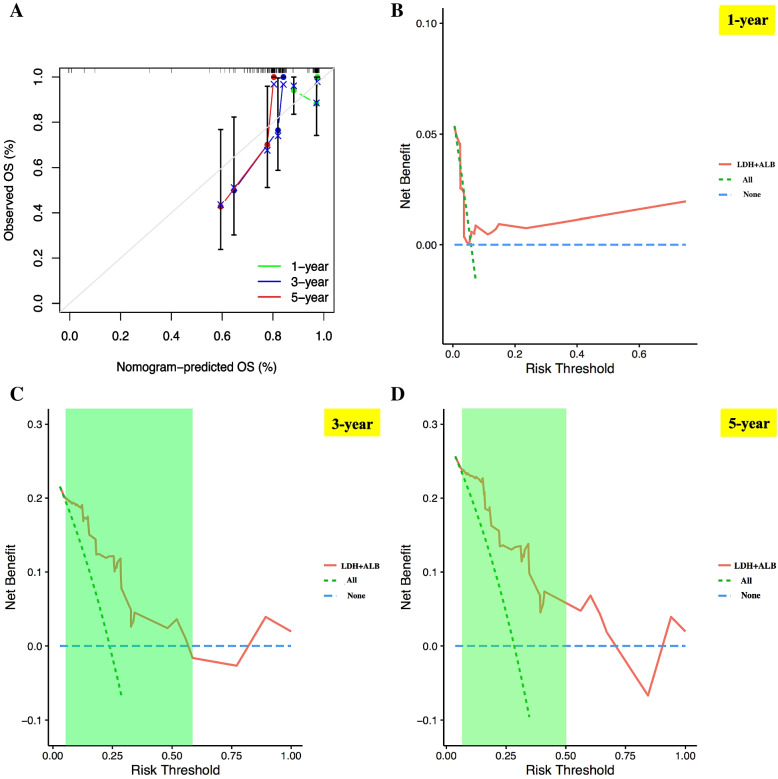
Verification of the nomogram discriminatory performance in validation set. **A** calibration plot verifying the estimation accuracy of the 1-, 3- and 5-year OS rates based on the nomogram. **B**, **C** and **D** DCA curves verifying the clinical value of the nomogram model for predicting 1-, 3- and 5-year OS rates, respectively. It should be noted that for validating the clinical value of the model for 3- and 5-year OS rates, the net benefits were mainly determined by the sizes of area of right upper quadrant in green region. NB, neuroblastoma; OS, overall survival; DCA, decision curve analysis

**Fig. 5:**
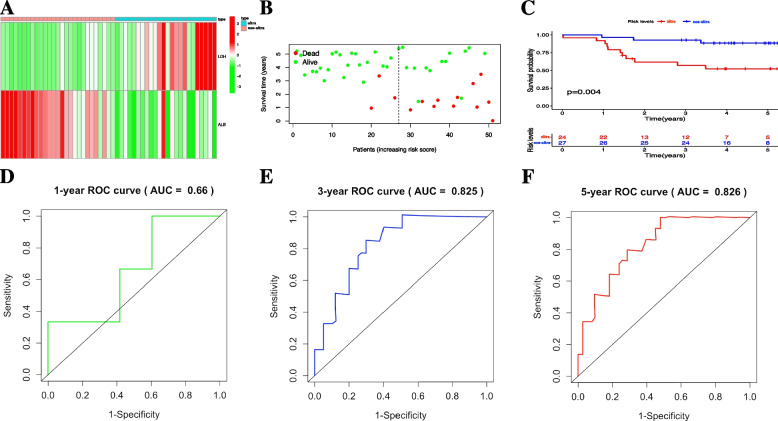
Verification of the nomogram prognostic accuracy in validation set. **A** serum LDH and ALB level heatmap, (**B**) survival time distribution plot and (**C**) the Kaplan–Meier curve verifying the nomogram prognostic accuracy between non-ultra high-risk and ultra high-risk groups in validation set. **D**, **E** and **F** ROC curves verifying the prognostic accuracy of the 1-, 3- and 5-year OS rates based on the nomogram. NB, neuroblastoma; OS, overall survival; LDH, lactate dehydrogenase; ALB, albumin; ROC, receiver operating characteristic

In the validation cohort, The C-index of nomogram model in validation set was 0.773 (95%CI: 0.664—0.897), which further confirmed that the nomogram model exhibited good discriminatory power in prognosis prediction of high-risk NB. Moreover, we conducted calibration plot analysis in validation set as demonstrated in Fig. 4A, the result showed that this nomogram model also had good probability consistencies between actual 1-, 3- and 5-years OS and nomogram-predicted 1-, 3- and 5-year OS. Similarly, applying this nomogram model to predict high-risk NB 3- and 5-year survival could gain more net benefits under the risk threshold of < 0.60 and < 0.50, as reflected by the result of DCA in validation cohort (area of right upper quadrant in green region in Fig. 4C, D). Moreover, for predicting 1-year OS rate in validation cohort, DCA curve also show much net benefit from nomogram compared with the hypothetical treat-none or treat-all scenarios (Fig. 4B). Based on the same cutoff risk score from nomogram applied for calculating the children’ nomogram score in training cohort (34 points), the pediatric patients in validation cohort (*n* = 51) were also clustered into two clusters: ultra high-risk cluster (*n* = 24, nomogram score > 34 points) and non-ultra high-risk cluster (*n* = 27, nomogram score ≤ 34 points) (Supplemental file 4). The LDH + ALB heatmap and the survival time distribution plot based on this nomogram model in validation set also demonstrated a wonderful predictive value (Fig. 5A, B). Kaplan–Meier survival analysis was performed and the result implied that non-ultra high-risk children had obvious better prognosis than ultra high-risk children (*p* = 0.004, Fig. 5C). AUC values of ROC curves for 1-, 3- and 5-year OS rates in the validation cohort were 0.660, 0.825 and 0.826, respectively, indicating that the nomogram model also had a good prognostic accuracy in the aspect of 3- and 5-year OS rates in validation set (Fig. 5D-F).

## Discussion

High-risk NB is the main risk type of NB with the high degree of cancer mortality rate [17]. It is needed to develop a readily available, low-cost and easy-to-use tool for clinical application to identify ultra high-risk NB patients, a subgroup of high-risk NB with the worst prognosis, and help guide novel and intensive front-line treatments earlier for them. In current study, we successfully constructed a nomogram based on Chinese population for predicting 1-, 3- and 5-year OS for the first time, and to identify those newly diagnosed high-risk NB individuals who are at the highest risk of death within the 5 years, by combining pretreatment blood-derived biochemical indicators, including LDH and ALB. ROC curve was performed to evaluate the prognostic accuracy of the nomogram model in training and validation cohorts. Model discriminatory ability was also assessed through calibration plot, Kaplan–Meier analysis and DCA curve in both cohorts, and the results revealed that the nomogram model had powerful prognostic potential and high-risk NB patients could make use of it clinically to predict their 3- and 5-year OS rates, enabling them to further understand their survival condition within the first 3 or 5 years of diagnosis before initiation of anti-cancer therapy. Most importantly, clinicians could apply it to identify potential ultra high-risk NB children at the time of diagnosis, offering them access to innovative anti-NB treatments early in their following course of multimodal therapies and aiding their clinical decision-making.

Currently, multiple efforts are attempting to improve our understanding of the tumorigenesis of NB and its upstream genomic aberrations [18]. For examples, *MYCN* amplification, genetic aberrations of chromosomes 1p, 11q and 17q, DNA ploidy and *ALK* mutation are the main known upstream genomic aberrations that play key roles in the NB tumorigenesis and are highly associated with NB prognosis. Therefore, these upstream genomic aberrations had been successfully utilized in risk stratification. Unfortunately, hospitals in many low- and middle-income countries or regions such as western provinces of China, where professional tumor pathologic examination may be unavailable, and technical or financial resources may be also not enough to perform genomic analysis of ploidy, genetic aberrations of chromosomes and *MYCN* status. Thanks to the heterogeneity of NB biology that provides us a large number of clinical and biological indicators with potential prognostic values, including not only upstream factors of genomic aberrations, but also downstream indicators of clinical manifestations, opening the door for consideration and testing of many candidate downstream clinical biofactors. That is a great opportunity to apply these easy-to-obtain and low-cost downstream clinical biofactors especially in low- and middle-income countries or regions to predict survival outcome of high-risk NB.

In the current study, incorporated prognostic variables—LDH and ALB—are all easily obtained and low-cost downstream clinical biofactors known for the vast majority of patients in the world. Among them, serum LDH is a common biomarker of tissue damage and has been found to be involved in multiple tumor biological processes, including cancer initiation [19], cancer cell invasion and tumor metastasis [20]. LDH also has been known as a prognostic biofactor in NB since 1992 [21], and its prognostic strength was emphasized recently by Moroz et al. [22], based on a large NB cohort. These studies have indicated that high level of LDH in serum could play as an independent prognostic biofactor for NB patients in predicting OS, and was highly associated with poor OS, which was also reflected in our study. Nutritional status has been found to be highly related to tumor initiation and development in children [23]. Serum ALB, one of the important nutrition-related indicators, was listed in our nomogram. Hypoalbuminermia is a common clinical event in cancer children and correlated with poor survival in many types of cancers [24]. In our study, we demonstrated that decreased ALB in serum was an independent prognostic indicator in high-risk NB for predicting OS. This might be due to the high aggressiveness and early extensive metastatic characteristics of high-risk NB, especially peritoneum metastasis with severe ascites. We therefore propose a hypothesis that ALB infusion may have therapeutic effects for improving the survival of high-risk NB children with severe hypoalbuminermia. However, Due to lack of prospective clinical study, whether an apparent increase in serum ALB levels could improve prognosis of high-risk NB children with severe hypoalbuminermia still remains undetermined. We hope our findings in this study will stimulate future prospective clinical studies on exploring the use of ALB infusion in anti-NB treatments for high-risk NB children with severe hypoalbuminermia.

On the other hand, the main reasons why we did not add *MYCN* status or chromosomes 11q aberration to classify ultra high-risk NB in this study can be summarized as follows: (1) Firstly, this study mainly aimed to develop a survival prediction nomogram that could be used for high-risk NB children in developing countries or regions that lack of necessary technical or financial resources to perform genomic analysis. Based on this point, it seems that there is no need to add *MYCN* status or chromosomes 11q aberration indictors to construct prognostic nomograms in current research. (2) Secondly, from the patients' view, many patients' parents, including many recruited patients' parents in this study, are unwilling to choose these genomic tests (detections of *MYCN* status and 11q aberration) due to the expensive costs of fluorescence in situ hybridization (FISH) technology and relatively hard-to-obtain sample requirements (fresh tumor specimens or bone marrow samples). Consequently, the *MYCN* status or chromosomes 11q aberration of many patients in current study were unknown or unavailable before treatment, which obviously affected the predictive performances of these genomic analysis indicators in our cohort. (3) Finally, there have already been a study designed by Moreno L et al., which included *MYCN* status indicator to construct a survival prediction nomogram for high-risk NB children [3]. Based on their study, it is meaningful for us to develop a nomogram without genomic indicators and explore the predictive difference between their nomogram model and ours. The nomogram we established and validated based on the real-world population might represent a promising survival prediction tool for high-risk NB children at their pretreatment stage. According to the 3-year OS nomogram of high-risk NB constructed by Moreno L et al. [3], using indicators of *MYCN* status, serum LDH and presence of bone marrow metastases, the AUC value of their validation cohort from SIOPEN HR-NBL1 trails was reported to be 0.629, while the AUC of our 3-year OS nomogram in current training and validation sets was 0.758 and 0.825, respectively. Our model showed enhanced prognostic accuracy in predicting 3-year OS for high-risk NB children when compared with the nomogram model created by Moreno L et al., although the numbers of individuals enrolled in current research was relatively small that could cause unpredictable bias. Therefore, a larger patient population from other cancer center should be included to further validate our nomogram. Besides that, our prognostic nomogram was based on a retrospective analysis. How it acts in prospective researches remained to be further evaluated. Moreover, our nomogram model demonstrated specificity for Chinese high-risk NB patients, whether it could also be applied to other risk types of NB and other race of high-risk NB remained to be further determined.

## Conclusions

In summary, we developed and validated an easy-to-use pretreatment nomogram based on common and low-cost blood-derived biofactors—LDH and ALB—for mainly predicting 3- and 5-year OS in high-risk NB patients. It might help patients estimate the survival condition in their first 3 and 5 years of diagnosis and select appropriate anti-NB therapy options, and help clinicians identify the potential ultra high-risk NB subgroup with particularly low rate of 3- and 5-year OS before treatment so that they could provide those children even from developing countries or regions access to innovative or intensive multimodal treatments earlier in the disease course.

### Supplementary Information

**Additional file 1:** **Supplementary file 1.** Detailed information about clinical characteristics of the high-risk NB children in training set.

**Additional file 2:** **Supplementary file 2.** Detailed information about clinical characteristics of the high-risk NB children in validation set.

**Additional file 3:** **Supplementary file 3.** Detailed information about the risk stratification of the high-risk NB children based on nomogram model in training set.

**Additional file 4:** **Supplementary file 4.** Detailed information about risk stratification of the high-risk NB children based on nomogram model in validation set.

## Data Availability

The raw data of this study are available from additional files (Supplementary files 1 and 2). Further inquiries can be directed to the corresponding authors via email.
